# Biomechanical analysis of the closed reduction internal fixation with cannulated screw of femoral neck fractures

**DOI:** 10.1097/MD.0000000000024834

**Published:** 2021-02-26

**Authors:** Jian-Guang Wang, Jun-Xian Wu, Yu-Min Li, You-Yin Xu

**Affiliations:** Department of Orthopedics, Civil Aviation General Hospital, No.1, Gao Jing Jia Chao Yang Street, Chaoyang District, Beijing, People's Republic of China.

**Keywords:** femoral head necrosis, femoral neck fracture, logistic regression model analysis, related factors

## Abstract

The influencing factors in closed reduction internal fixation with cannulated screw of femoral neck fractures have not been well investigated. This study evaluated these factors in patients with femoral neck fractures.

Fifty-seven patients (36 males and 21 females) diagnosed with femoral neck fracture with the average age of 52.44 ± 15.04 years who underwent closed reduction internal fixation with cannulated screw were included in this study. Data were collected through case report reviews, phone call follow-ups, and outpatient follow-ups to evaluate pre- and postoperative radiograph images. Statistical analysis was performed using Garden classification, binary and multinomial logistic regression analysis by including factors such as patient's age, gender, fracture type, time to fixation, reduction quality, functional recovery period, removal of cannulated screw, and preoperative traction. Logistic regression analysis revealed that age and reduction quality was statistically significant (*P* < .05) to clinical outcome and other factors were not statistically significant.

The main factors affecting clinical outcomes were functional recovery and reduction quality. The biomechanical effects of fixation provide a good foundation for fracture healing. Patient's conditions should be carefully evaluated before selecting reduction procedures to reach an optimal surgical outcome.

## 1 Introduction

A femoral neck fracture is a common orthopedic trauma condition due to increasing traffic collisions and the aging population. Surgical fixation for femoral neck fracture includes internal fixation with cannulated screw and hip replacement. Internal fixation with cannulated screw is most frequently used in treating femoral neck fractures for it can achieve satisfactory clinical results as well as increase fracture healing rate.^[1,2]^ It is the best treatment option for young patients with nondisplaced femoral neck fractures. Studies have shown the incidence of complications such as osteonecrosis being 20% to 30% and fixation failure being 8% to 15%, respectively.^[3]^ Compared with non-displaced femoral neck fracture, displaced fracture of the subcapital femoral neck are more likely to cause complications, such as aseptic necrosis or nonunion.^[4–7]^ Every year, in the US, 650,000 female patients die from complications as a result of hip fracture, and only 25% of the patients can recover to preoperative conditions.^[8,9]^ Early anatomical reduction and stable internal fixation can effectively prevent complications such as osteonecrosis and nonunion, especially in young patients.^[10–12]^

There is currently a lack of study on the effect and correlation factors for internal fixation with cannulated screw of femoral neck fractures. We conducted a retrospective study on 57 cases of femoral neck fractures. Attention was given to the effect and influencing factors of internal fixation with cannulated screw of femoral neck fractures through logistic regression analysis, combined with biomechanical analysis. The aim is to determine the main factors that can affect clinical outcomes of internal fixation with cannulated screw.

## 2 Materials and methods

### 2.1 Methods

From August 2010 to October 2017, 57 patients (36 males and 21 females) diagnosed with femoral neck fracture with the average age of 52.44 ± 15.04 years were treated. Grouped by the Garden classification, there were 13 type II, 33 type III, 11 type IV fractures. All patients were treated with closed reduction internal fixation with cannulated screw. We utilized case report reviews, phone call follow-ups, and outpatient follow-ups to evaluate pre- and postoperative radiograph images in a comparative analysis; Magnetic resonance imaging (MRI) to detect osteonecrosis if required. We also assessed factors such as patient's age, gender, fracture type, time to fixation, reduction quality, functional recovery period, removal of cannulated screw, and preoperative traction. All patients provided signed informed consent, and the study was approved by the institutional review board.

### 2.2 Surgical technique

All patients underwent surgery by closed reduction internal fixation with cannulated screw. After performing epidural anesthesia and C-arm fluoroscopy X-ray, Garden classification type I to II was used [angle of compression trabeculae of the femoral shaft and head on anteriorposterior (AP)] view was 155 to 160 degrees; femoral head and neck axis were co-linear on lateral view) while maintaining reduction position. The patient's pelvic was padded to achieve 15 degrees tilt (easier to operate) and routine disinfection and draping were performed. The percutaneous guiding needle was inserted from the greater trochanter into the subtrochanteric region, subsequently, under the guidance of C-arm, 3 guiding needles were inserted 0.5 cm beneath the femoral head. While keeping the neck shaft angle and anteversion angle in mind, the three guiding needles formed an “inverted triangle” with the lateral and medial cortex in the subtrochanteric region at the same time keeping the 2 guiding needles on a parallel axis without intersecting (otherwise screw cannot be implanted). Once the guiding needles were in position, 1 cm incisions were made into the cortex of the subtrochanteric region. After measuring the depth, drill sleeves were used to protect the drill and taps as cannulated screws with according length were placed. Padding was added to the base of the screw if basal neck fracture was present. The incision was sewn closed after checking with C-arm and removing guiding needles. Routine lateral postoperative radiographs were given at each follow-up.

### 2.3 The reduction acceptability assessment

The anatomical reduction was assessed by using the Garden alignment index and lateral radiographs to classify fractures into 4 types. Type I: lateral view falls within 155 to 180 degrees; Type II: at least one, AP or lateral view, is less than 155 degrees or 180 degrees; Type III: AP view is both less than 155 degrees and lateral view is more than 180 degrees; Type IV: AP view is less than 155 degrees or lateral view is more than 180 degrees. Type I and II is a good outcome, Type III and IV is a poor outcome.

### 2.4 Statistical analysis

According to previous studies and clinical results, 8 factors that affect clinical outcomes were selected. Each factor was assigned and quantized. All statistics were performed using SPSS 22.0 software to carry out binary and multinomial logistic regression analysis. A *P*-value < .05 is considered as statistically significant (Table 1). All data were entered into EXCEL; the statistical analysis included binary and multinomial logistic regression analysis. All statistics were performed using SPSS 22.0 software.

**Table 1:** Quantitative evaluation of related factors of avascular necrosis of the femoral head.

Factors	Variables	Assigned Values
Union of fracture	Y	No = 0, Yes = 1
Gender	X1	Male = 0, Female = 1
Age, yr	X2	≤50 = 0, >50 = 1
Time to fixation, d	X3	≤3 = 0, >3 = 1
Preoperative traction	X4	No = 0, Yes = 1
Reduction quality	X5	Nonanatomical reduction = 0, Anatamical reduction = 1
Functional recovery period (months)	X6	3∼6 = 0,>6 = 1
Garden classification	X7	1,2 = 0,3,4 = 1
Removal of cannulated screw	X8	No = 0,Yes = 1

## 3 Results

### 3.1 Result of statistical analysis

The assigned and quantized effect and influencing factors are summarized in Table 1. By applying binary logistic regression analysis to these factors, we discovered that age and reduction quality was statistically significant (*P* < .05) to clinical outcome and other factors were not statistically significant (Table 2). Multinomial logistic regression analysis revealed the risk factors for femoral neck fracture included age and reduction quality (*P* < .05) summarized in Table 3.

**Table 2:** Analysis results of single factor logistic regression model.

Factors	Variable	B	SE	W	*P*	Exp (B)
Gender	X1	−0.530	1.350	0.154	.694	0.589
Age	X2	0.014	0.066	0.046	.831	1.014
Time fixation	X3	−0.004	0.010	0.128	.720	0.996
Preoperative traction	X4	−0.533	1.401	0.145	.704	0.587
Reduction quality	X5	−3.798	1.605	5.598	.018	0.022
Functional recovery period	X6	1.593	0.771	4.268	.039	4.918
Garden classification	X7	−0.078	1.035	0.006	.940	0.996
Removal of cannulated screw	X8	0.124	1.251	0.010	.921	1.132

**Table 3:** Analysis results of multivariate factor logistic regression model.

Factors	Variable	B	W	*P*	Exp (B)
Gender	X1	−0.396	0.042	.837	0.673
Age	X2	0.085	0.906	.341	1.089
Time fixation	X3	−0.001	0.014	.905	0.999
Preoperative traction	X4	1.691	0.976	.323	5.422
Reduction quality	X5	28.162	10.230	.001	5.686
Functional recovery period	X6	33.485	15.553	.016	21.491
Garden classification	X7	−0.219	0.022	.882	0.803
Removal of cannulated screw	X8	−1.881	1.059	.303	0.152

## 4 Discussion

Due to the existence of neck shaft angle and anteversion angle, forces sustained are a combination of compressive stress, tensile stress, bending stress, and shear stress. The internal fixation apparatus can counter rotation stress and shear stress. It is easy to maneuver and causes minimal damage to the cortex of the femoral neck. By using cannulated screw to treat femoral neck fracture, the 3 screws are assembled in an edge scattering distribution that creates an “inverted triangle,” which opposes shear stress on the cut bone and increases the stability of internal fixation. Stable internal fixation can be achieved through 3-point fixation, the effect of an internal splint and compressing bone fragments to promote fracture healing.

The application of cannulated compression screw is originally used in the 1980s to perform internal fixation of femoral neck fractures. Previous studies have shown that when using closed reduction internal fixation with cannulated screw for femoral neck fractures, it does not reduce the occurrence rate of osteonecrosis, but the outcomes remain optimal.^[13,14]^ In this study, logistic regression analysis was performed to examine the eight possible effect and influencing factors associated with femoral neck fracture. Our findings indicate that closed reduction internal fixation with cannulated screw has a significant association with age and reduction quality; the biomechanical effect of fixation also provides a good basis for fracture healing.

Adequate reduction includes correct anatomical alignment and union of the fracture. Beris et al^[15]^ reported that the primary factors affecting the prognosis of femoral neck fracture is the quality of fracture reduction, subsequently, the structural stability. Hence, reduction quality and mechanical stability play a significant role in osteonecrosis in the postoperative femur.^[16]^ In our study, age is an important influencing factor in the clinical outcomes. The cause for femoral neck fracture is different in younger and elderly patients in which the prior is usually caused by high-energy trauma, latter is by osteoporosis. Dai et al^[17]^ performed meta-analysis on elderly patients with displaced femoral neck fracture who underwent femoral replacement or internal fixation, and when the 2 procedures were compared, femoral replacement has a lower probability of secondary surgery and the occurrence of complications. They concluded that femoral replacement is a better treatment option and there is no statistical significance between either procedure to a 1-year mortality rate. In healthy elderly patients with long life expectancy and the prospect of achieving anatomical reduction, closed reduction internal fixation with cannulated screw remains the best surgical option.

The stability of internal fixation directly affects fracture reduction and the occurrence of complications; the distribution of the screws after fixation is closely linked to the femoral neck's biomechanical stability. The biomechanics should be carefully evaluated before implantation and distribution of the screws. Neck shaft angle and anteversion angle formed by the femoral neck and shaft generate compressive stress on the medial aspect of the femoral neck and minor tensile stress on the lateral aspect. The femoral neck also sustains shear stress, and thus, the internal fixation apparatus not only needs to counter rotation stress and shear stress, but it must also be easy to maneuver and creates minimal damage to the cortex of the femoral neck. The use of the “inverted triangle” fixation model consists of 2 screws above the neutral axis of the femoral bending, where the regular triangle only has 1 screw. Hence, the “inverted triangle” has a stronger bending modulus, less flexural deformation, and better stability than the regular triangle. The 3 screws of the “inverted triangle” model are distributed evenly and have an internal splint effect to provide 3-point support to the cortex, medulla, and femur head. This mechanism opposes shear stress on the surface of the cut bone; prevents displacement inferiorly and anteriorly; minimizes displacement after internal fixation. Stable internal fixation creates a good basis for fracture healing, decreases nonunion, and lowers the probability of osteonecrosis.

## 5 Conclusion

Our study utilized multinomial logistic regression analysis and findings suggest that when using internal fixation with cannulated screw, functional recovery, and reduction quality are the main factors that affect the clinical outcomes of femoral neck fracture. The biomechanical effect of fixation is crucial to our study. Furthermore, other influencing factors such as fracture type and removal of cannulated screw also affect the union of the fracture, but it was not highlighted in our study due to a lack of sample size.

Our study has a relatively small sample size and we postulate that bigger sample size is required to further investigate how the influencing factors and biomechanical effect of fixation can affect the treatment options for femoral neck fractures.

## Author contributions

**Supervision:** Jian-Guang Wang.

**Writing – original draft:** Jian-Guang Wang.

**Writing – review & editing:** Jian-Guang Wang, Jun-Xian Wu, Yu-Min Li, You-Yin Xu.
